# Determinants of COVID-19 Mortality and Temporal Trends in the Health Regions of the State of São Paulo, Brazil

**DOI:** 10.3390/ijerph22050772

**Published:** 2025-05-13

**Authors:** Tatiana Pestana Barbosa, Thais Zamboni Berra, Antônio Carlos Vieira Ramos, Yan Mathias Alves, Reginaldo Bazon Vaz Tavares, Fernando Spanó Junqueira de Paiva, Jonas Bodini Alonso, Titilade Kehinde Ayandeyi Teibo, Juliana Soares Tenório de Araújo, Ariela Fehr Tártaro, Ricardo Alexandre Arcêncio

**Affiliations:** 1 Department of Maternal-Infant and Public Health Nursing, Ribeirão Preto College of Nursing, University of São Paulo, Ribeirão Preto 14040-902, Brazil; thaiszamboni@live.com (T.Z.B.); yan.alves@usp.br (Y.M.A.); reginaldobazon@usp.br (R.B.V.T.); jonasbal@eerp.usp.br (J.B.A.); tayandeyi@usp.br (T.K.A.T.); julianastenorio17@gmail.com (J.S.T.d.A.); ariela.ft@usp.br (A.F.T.); ricardo@eerp.usp.br (R.A.A.); 2 Department of Nursing, State University of Minas Gerais, Passos Campus, Passos 37900-106, Brazil; antonio.ramos@uemg.br; 3 Polytechnic School, University of São Paulo, São Paulo 05508-010, Brazil; fernandosjp@gmail.com

**Keywords:** COVID-19, mortality indicators, epidemiology

## Abstract

**Background:** This study investigated the determinants of COVID-19 mortality and its temporal trends within São Paulo state’s *Departamentos Regionais de Saúde* (DRS) (health regions) to inform the development of targeted public health interventions. **Methods:** Utilizing an ecological study design, we analyzed confirmed COVID-19 cases and deaths (February 2020–December 2021) obtained from the COVID Panel, incorporating relevant social and health indicators. The Generalized Additive Model for Location, Scale, and Shape (GAMLSS) was used to identify key determinants, and temporal trends in mortality and vaccination rates were analyzed across each DRS. **Results:** The average mortality rate was 15.1 deaths per 100,000 inhabitants (median 7.00). Higher chronic disease mortality was associated with an increase in COVID-19 mortality. Moreover, an increase in the percentage of adults led to a decrease in deaths from COVID-19. **Conclusions:** COVID-19 mortality in São Paulo is shaped by a complex interplay of 12 behavioral, economic, demographic, and environmental factors. Region-specific public health policies should consider these factors, along with geographic, socioeconomic, and budgetary contexts, to effectively address health disparities across the state’s DRS.

## 1. Introduction

COVID-19 has emerged as one of the most devastating public health crises of the 21st century, leading to numerous fatalities and widespread suffering globally [1]. The course of this pandemic can be characterized by distinct phases, starting from a period of limited understanding regarding the etiopathogenesis of the disease, progressing to the acquisition of epidemiological knowledge, and culminating in the formulation of public policy recommendations [2].

The disease had major impacts on the structure and organization of health systems and services around the world because the need for investments and reorganization of services to face COVID-19 brought losses in meeting other health demands [3]. Moreover, regions characterized by higher levels of social inequalities have experienced greater difficulties in managing the disease, having high incidence and mortality rates [1].

During the peak transmission period of COVID-19, various policies were implemented to mitigate the spread of the virus [2]. These measures included promoting social mobility restrictions, physical distancing, lockdowns, and, more recently, the widespread availability of COVID-19 vaccines, accompanied by efforts to expand vaccination [2].

Brazil established a universal health system in 1988 through its constitution, which defined health as a right for all citizens and a duty of the state. This system, known as the Sistema Único de Saúde (SUS), is one of the world’s largest public health systems. SUS guarantees free and comprehensive healthcare at all levels—primary, secondary, and tertiary—to the entire population.

The system is based on the principles of universality, equity, and integrality, ensuring that all residents, regardless of socioeconomic status, have access to health services. This commitment has led to significant improvements in health outcomes, such as increased life expectancy, a dramatic reduction in infant mortality, and the rapid expansion of COVID-19 vaccine coverage, which helped overcome the crisis brought on by the pandemic [2].

Specifically in the first two years of the pandemic, Brazil implemented various strategies to control COVID-19 [4]. Among these, the strategy used in São Paulo, the most economically developed state with the largest population in Brazil, stands out and was called “Contingency Plan of the State of São Paulo for Human Infection by the new Coronavirus—SARS-CoV-2”.

In short, the São Paulo Contingency Plan was prepared and constantly updated in relation to the state’s response level to COVID-19 and aspects related to the management of the outbreak, involving the establishment of sanitary measures, epidemiological commitments and the organization of services for the execution and follow-up of planned pandemic response actions [4]. The São Paulo State Contingency Plan used the division of the state into 17 *Departamentos Regionais de Saúde*—DRS (Health Regions) to monitor and compare the spread of diseases [5].

Historically, since the establishment of SUS, São Paulo state has organized its health services by Health Regions to promote justice and equity in healthcare access. Each one is designed to centralize essential resources—such as healthcare facilities, professionals, and services—to meet the health needs of its local population [4].

These Health Regions serve as the central hubs for planning, management, and delivery of health services, bringing together resources from federal, state, and municipal levels, fostering collaboration and co-management through regional commissions and agreements. This structure not only improves the distribution of health resources but also reduces regional disparities and strengthens the SUS’s capacity to respond to public health challenges and catastrophes, including COVID-19 [6]. Given its economic, social and political diversity, the state of São Paulo exhibits significant heterogeneity in terms of COVID-19 transmission [7].

Analyzing data across the entire territory tends to generate incorrect conclusions and, for this reason, working based on Health Regions becomes more appropriate since they have some particularities regarding the tradition of building regionalized policies, the dynamics of population movement, geographical characteristics, socioeconomic conditions, budgetary conditions and participation in the different Regional Health Care Networks.

Political and management strategies are essential in addressing the challenges arising from COVID-19 and ensuring the efficient functioning of the health system. Although this topic is of paramount importance for strengthening the SUS, no studies were found in the literature that have addressed the subject from the perspective of the Health Regions in São Paulo and consider the repercussions of the disease in the state. Thus, this study aimed to identify the determinants of COVID-19 mortality and temporal trends according to health regions of the state of São Paulo.

## 2. Materials and Methods

This is an ecological study conducted in São Paulo state, located in the southeast region of Brazil [8]. São Paulo state encompasses 645 municipalities and, in 2021, had an estimated population of 46,649,132 inhabitants, with a population density of 186.48 inhabitants/km^2^ [8].

**Figure 1:**
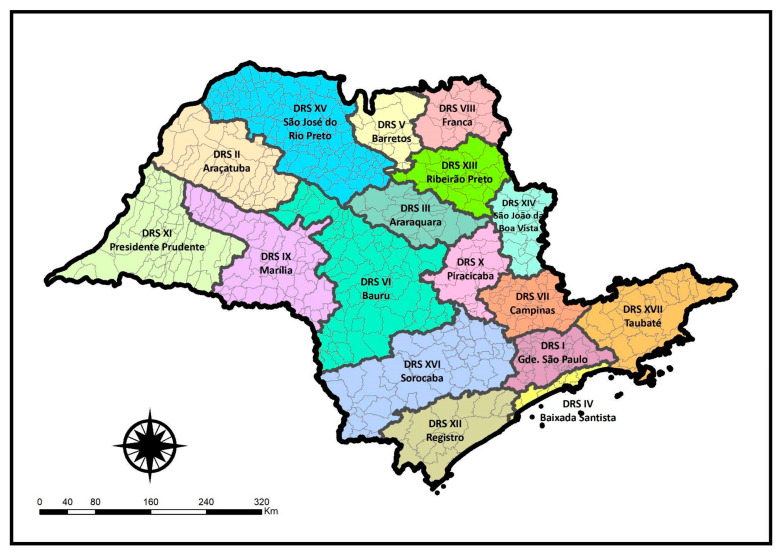
Health Regions in the state of São Paulo.

The state of São Paulo is administratively divided into 17 DRS, each covering all municipalities in the state. For this study, the unit of analysis was the subdivision of the state into these 17 DRS regions, as depicted in Figure 1: DRS I—Grande São Paulo, DRS II—Araçatuba, DRS III—Araraquara, DRS IV—Baixada Santista, DRS V—Barretos, DRS VI—Bauru, DRS VII—Campinas, DRS VIII—Franca, DRS IX—Marília, DRS X—Piracicaba, DRS XI—Presidente Prudente, DRS XII—Registro, DRS XIII—Ribeirão Preto, DRS XIV—São João da Boa Vista, DRS XV—São José do Rio Preto, DRS XVI—Sorocaba e DRS XVII—Taubaté.

The study period was from January 2020 to December 2021, allowing for an assessment of unique, first- and second-dose vaccine coverage [9], and which coincided with the initiation of vaccination efforts (the nationwide COVID-19 vaccination campaign began in January 2021).

Data collection on mortality from COVID-19, along with health and social indicators, was conducted in April 2022. COVID-19 data were obtained from the State System Foundation for Statistical Data Analysis (SEADE) [10]; vaccination data came from DATASUS (Department of Information and Health Informatics of SUS) [11]; and population data were sourced from the Brazilian Institute of Geography and Statistics (IBGE) [8]. The Gini index, sourced from DATASUS, according to the World Bank, is a measure of income or consumption distribution within an economy. It indicates the extent to which the distribution deviates from perfect equality, with a Gini index of 0 representing perfect equality and an index of 1 indicating perfect inequality. Table 1 presents the variables collected and their respective data sources.

The inclusion criteria specified confirmed COVID-19 deaths between January 2020 and December 2021, encompassing all municipalities and residents (urban and rural) and analyzing general trends rather than seasonal variations. Mortality rates were calculated by dividing the number deaths by the population of each municipality (using population estimates for 2021).

The initial step in preparing the database for subsequent analyses involved transforming the categorical variable representing different regions into a factor, enabling appropriate analysis within the model. To address missing values, an assessment of the dataset was conducted, identifying and imputing missing values in key variables using their respective mean values. This step helped maintain the integrity of the analysis by preventing gaps in the data.

Normalization of continuous variables was performed to scale all variables similarly, resulting in a standardized dataset. This transformation is crucial in statistical modeling, ensuring that no single variable disproportionately influences the results. Furthermore, dummy variables were created for categorical data, allowing the model to effectively leverage the information contained within these variables. The dataset was then filtered to focus on records from a specified time period, further refining the data for analysis.

Data analysis included exploratory descriptive analyses (minimum, maximum, mean, median, and boxplots).

For a given real number λ ≠ 0, the Box–Cox transformation *T*_*λ* is defined as follows:

*T_λ*(*x*) = (*x^λ* − 1)/*λ*

where *x* is a positive variable (*x* > 0).

In the special case where *λ* = 0, we have the following:

*lim* (*λ* → 0) [(*x*^*λ* − 1)/*λ*] = *log*(*x*)

This limit allows us to define the following:

*T*_0(*x*) = *log*(*x*)

where we consider the logarithmic transformation as a special case of the Box–Cox transformation. In this analysis, a constant was first added to the mortality rate to avoid zeros, specifically by incrementing the values by 1. The parameter (*λ*) was subsequently determined through the Box–Cox [12,13] method to achieve optimal transformation of the data, which was then used in further analyses.

The GAMLSS [14] model was structured using this distribution, along with an appropriate link function. The probability associated with the Gamma distribution is utilized, as the transformed mortality rate showed no significant outliers and presented a tendency favoring this distribution. With this setup, the model became capable of estimating COVID-19-related mortality more accurately, taking into account the inherent variability in the data.

The aim of this analysis was to investigate the association between the COVID-19 mortality rate (dependent variable) and social and health indicators (independent variables) at the DRS municipal level in the state of São Paulo.

The GAMLSS model class is especially suitable for modeling the dependent variable due to its analytical flexibility and the wide range of groups of probability distributions available. This model allows for up to four different parameters to be employed in modeling the dependent variable, enabling a better adjustment to different datasets [13].

The model consists of a vector *yT* = (*y*1, …, *yn*), with a size of n, representing the dependent variable. It has a density function *f*(*yi*|*θi*), where *θi* = (*θ*1*i*, *θ*2*i, θ*3*i, θ*4*i*) = (*σi, νi, τi*), and *k* = 1, 2, 3, 4. The function *gk*( ) represents a monotonic link function that relates the parameters to the independent variables of the following equations:

*g*1(*µ*) = *η*1 = Χ1β1 + ∑*h*j1 J1 j = 1 (xj1)

*g*2(*σ*) = *η*2 = Χ2β2 + ∑*h*j2 J2 j = 1 (xj2)

*g*3(ν) = *η*3 = Χ3β3 + ∑*h*j3 J3 j = 1 (xj3)

*g*4(τ) = *η*4 = Χ4β4 + ∑*h*j4 J4 j = 1 (xj4)

where *µ*, *σ*, ν and τ are vectors of length *n*, *βk* = (*β*1*k*, *β*2*k*, …, *β*j*k*) is a vector of length *jk*, and *Xk* is the design matrix of order *xjk*. Function *hjk* is a non-parametric non-additive function of the explanatory variable *Xk* evaluated in *xjk*.

The selection of the distribution of the dependent variable was based on the Generalized Akaike Information Criterion (GAIC), which is defined as GAIC = −2l(*θ*) + *bdf*, where l(*θ*) is the likelihood function, b is a penalty parameter and df denotes the degrees of freedom of the model. For the original Akaike Criterion (AIC), b is set to 2 [15].

The choice of the Gamma distribution for the GAMLSS model was based on the nature of the data, which exhibited positive skewness and was strictly non-negative.

The GAMLSS model was structured using the identity link function. The probability density function associated with the Gamma distribution was utilized, as the transformed data exhibited a tendency to follow this distribution. The expected value of the random variable (*Y_t*) is simply represented by the estimated means, given that the Gamma distribution has the property that its expectation is determined by the shape and scale parameters, rather than a single location parameter as in the normal distribution [16].

Based on the selected GAMLSS model (Gamma), the Relative Increase (AR) of the COVID-19 mortality rate was calculated as a percentage (%), using the expression *AR*(*β*) = exp(*β*) − 1 × 100%.

The adequacy of the model can be checked through the model’s diagnosis charts, including Adjusted Values x Residuals, Order of Observations x Residuals, Distribution of Residuals and Quantile Quantile Graph (Q-Q plot). Additionally, the Kolmogorov–Smirnov normality test was applied to the model residuals to verify their adequacy with the standard normal distribution.

Furthermore, a historical series chart was constructed for each DRS, comparing the behavior of deaths, mortality rate and COVID-19 vaccination over the year of investigation.

As the study utilized publicly available secondary data, it was approved by a Research Ethics Committee. To carry out all the analyses, the RStudio program version 4.2.1 (PBC, Boston, EUA) was used.

## 3. Results

Between January 2021 and December 2021, the estimated average mortality rate from COVID-19 was 15.10 deaths per 100,000 inhabitants, with a median of 7.0 per 100,000 inhabitants, as shown in Table 2 below.

We observed that the population aged between 15 and 60 years old corresponded to an average of 68.91% and a median of 65.38% of the total population. The urban population in the state of São Paulo represented, on average, 89.33%. The Gini index, which measures income distribution, had an average value of 0.4586, with a median very close to 0.473, indicating moderate income inequality in the state.

In terms of COVID-19 vaccination coverage, the total number of unique doses and the first and second doses of vaccination administered amounted to 4,314,565. This represents an average of 18,023.61 doses administered per segment of the population vaccinated. Table 2 presents the detailed results of the descriptive analysis conducted in the study.

**Figure 2:**
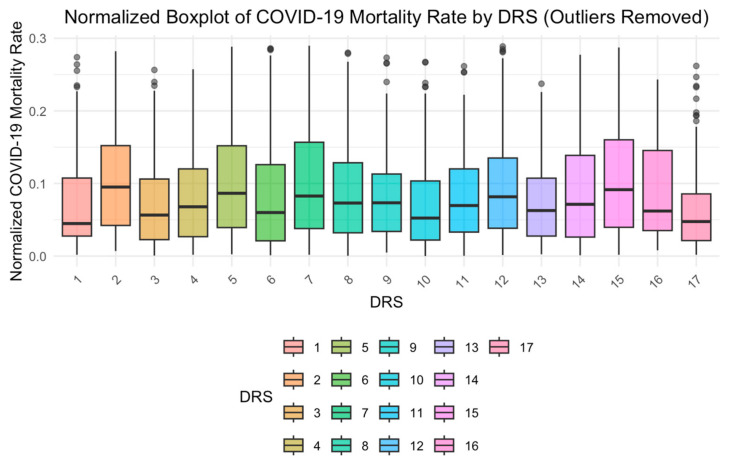
Normalized boxplot of COVID-19 mortality rates by DRS (outliers removed) (2021).

Figure 2 displays the distribution of the number of COVID-19 deaths according to DRS in the state of São Paulo. The regions with the highest number of deaths were as follows: DRS II—Araçatuba, DRS V—Barretos, DRS VII—Campinas, and DRS XV—São José do Rio Preto. To ensure a more robust analysis and to improve the clarity and interpretability of the visualizations, the interquartile range (IQR) method was used, while outliers in the normalized mortality data were identified and filtered out.

Regarding the GAMLSS result, for the 2021 period, variables such as deaths from chronic diseases (V2), and the percentage of the population aged 15–60 years (V4), demonstrated statistical significance, as shown in Table 3.

It was observed that a 1% increase in deaths from non-communicable diseases (NCDs) was associated with a relative increase of 8.62% in the mortality rate from COVID-19. Additionally, for each 1% increase in the percentage of the adult population, a relative decrease of 17.59% in the COVID-19 mortality rate can be expected. Furthermore, it was identified that for every 1% increase in mortality in DRS VI, VII, and XV, a relative increase of 41.77%, 67.08%, and 52.96%, respectively, in the COVID-19 mortality rate can be expected.

**Figure 3:**
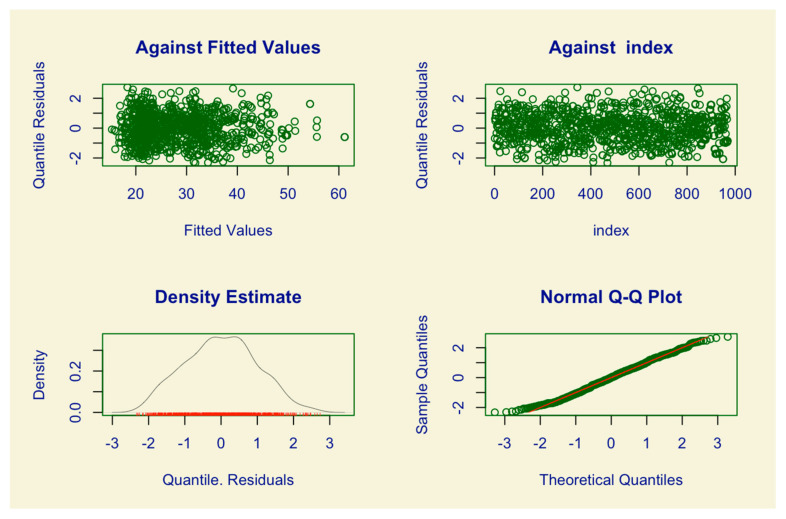
Diagnostic graphics of the adjusted model.

The Kolmogorov–Smirnov test, which was applied to the model’s residuals to test the assumption of normality, presented statistics D = 0.026509 and *p*-value = 0.5037. Based on these results, it can be concluded that the model fit was adequate, as the residuals followed a normal distribution. Figure 3 provides graphs that support the conclusion that the assumptions about the residuals of the presented model were satisfied, further confirming the model’s adequacy.

**Figure 4:**
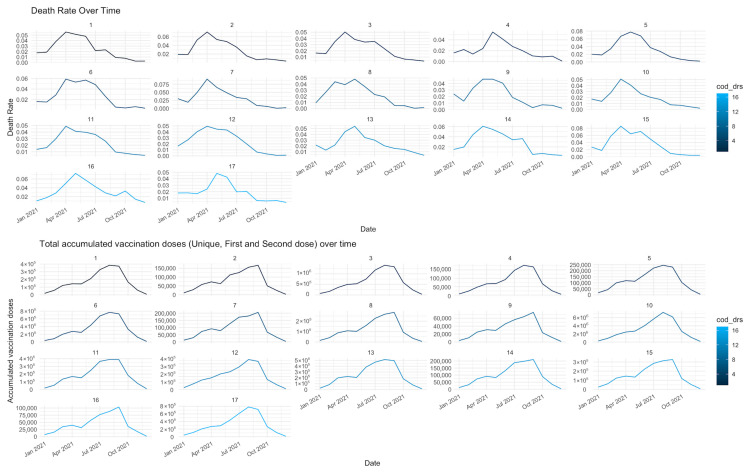
Time series analysis of COVID-19 mortality rates and total accumulated vaccination coverage (unique, first and second doses) across DRS.

Figure 4 demonstrates that in all DRSs, the mortality rates decrease with the increase in accumulated vaccination doses.

## 4. Discussion

This study aimed to identify the determinants of COVID-19 mortality and temporal trends across São Paulo state’s health regions. Using GAMLSS, we found that higher mortality from NCDs was associated with a higher COVID-19 mortality rate. Conversely, increases in the adult population were associated with lower COVID-19 mortality.

The analysis of COVID-19 mortality in the state of São Paulo revealed disparities in mortality across the state’s health regions, with distinct temporal trend patterns. These findings corroborate previous evidence on the heterogeneous burden of the disease in subnational contexts, where structural inequalities shape the impact of the pandemic [17,18]. Although organizing health services by health regions aims to promote justice and equity in healthcare access, the findings showed that this structure has failed to reduce regional disparities or even strengthen the SUS’s ability to respond to COVID-19, mainly in the social vulnerability contexts.

It was observed that health regions with higher social vulnerability exhibited elevated levels of mortality, even after the partial control of transmission in central urban areas. These findings reflect inequalities in the distribution of health services, testing capacity, and public health surveillance, particularly in peripheral and inland areas [19]. This pattern has also been documented in other middle-income countries, where the pandemic has exacerbated structural barriers to healthcare access [20].

With the introduction of vaccination in 2021, a uniform decline in COVID-19 mortality was expected across the state; however, the findings show that this trend was uneven among regions. Some regions with high vaccination coverage continued to report elevated mortality rates, especially during the first half of 2021. This association has also been reported in similar ecological analyses [21,22], and may reflect the prioritization of high-risk groups such as the elderly and individuals with comorbidities whose mortality rates remained high even after the start of vaccination [23].

In some regions, the persistence of high mortality rates even after the intensification of vaccination suggests the influence of contextual factors, such as unequal distribution of resources, low adherence to non-pharmaceutical interventions, and underreporting of mild cases. Additionally, increased public confidence in vaccines may have led to a premature sense of safety, resulting in reduced mask use and social distancing, a phenomenon previously described in other populations [24].

Furthermore, the effect of vaccines depends on the time window between the first dose, the completion of the primary regimen, and the development of protective immunity. Health Regions that experienced epidemic peaks before the full immunization of priority populations reported elevated mortality rates, which underscores the need for caution when interpreting correlations between vaccination and deaths in aggregate analyses [25].

The regional analysis allowed the identification of clusters with distinct epidemiological patterns, reinforcing the importance of territorialized epidemiological surveillance. Health Regions with a sustained downward trend in mortality, particularly from the second half of 2021, were regions that had an early start to elderly vaccination, hospital infrastructure, greater testing capacity, higher hospital bed density, and more robust laboratory systems.

These characteristics have been documented as key determinants of resilience during health crises [26,27]. In contrast, regions with rising trends or high fluctuations in mortality largely exhibited structural deficiencies and unfavorable social indicators—a pattern that underscores the importance of the intersection between epidemiology and territorial inequality [19].

The results of the GAMLSS model indicated that the mortality rate from NCDs was positively associated with COVID-19 mortality. Specifically, the model estimated that a 1% increase in NCD mortality corresponds to an 8.62% increase in COVID-19 mortality. NCDs—including systemic arterial hypertension, diabetes mellitus, cardiovascular diseases, and chronic respiratory diseases—are well-established risk factors for severe outcomes and death from COVID-19 [28,29]. Therefore, in population contexts with a higher burden of morbidity and mortality due to NCDs, increased COVID-19 mortality is to be expected, as observed in our study.

Regarding the population’s age structure, an inverse association was found between the proportion of adults (aged 15–60 years) and COVID-19 mortality: for each 1% increase in this age group, there was a corresponding 17% reduction in mortality. This finding may reflect the lower biological vulnerability of younger adults to severe outcomes of the disease compared to the elderly population [30].

Although the proportion of older adults did not show a statistically significant association in the final model, it is important to emphasize that elevated mortality among older individuals is well documented in the literature [31]. Therefore, regions with a higher proportion of adults may experience lower COVID-19 mortality rates, potentially due to the reduced risk of complications and death in this age group.

Additionally, analysis of the model revealed that higher mortality rates in the health regions of Bauru, Campinas, and São José do Rio Preto are linked to an overall increase in COVID-19 mortality across the various health regions of São Paulo State. This regional variability may reflect structural inequalities related to access to and quality of healthcare services, diagnostic capacity, epidemiological surveillance, as well as socioeconomic conditions and local responses to the pandemic.

Previous studies had already identified that the response to COVID-19 in Brazil was marked by significant regional disparities [32], and the results presented here reinforce this evidence by demonstrating that territorial context and the social determinants of health play a central role in shaping the burden of COVID-19 mortality, such that certain regions may have contributed disproportionately to the total number of deaths in the state. These findings underscore the importance of region-specific strategies aimed at mitigating health inequalities and strengthening surveillance and healthcare efforts in more vulnerable areas.

The limitations of this study include the risk of ecological fallacy, which is inherent in analyses based on aggregated data. The absence of individual-level information on vaccine type, the presence of specific comorbidities, and the interval between doses limits causal inferences. Furthermore, the potential underreporting of deaths and variations in data quality across regions may have introduced bias into the models. Future studies should consider using interrupted time series analysis to assess the impact of vaccination in contexts characterized by high epidemiological variability.

The findings have important implications for public health planning. The persistence of inequalities in COVID-19 mortality, even after the introduction of vaccines, underscores a crucial reality: while vaccination is a powerful and essential tool for reducing severe illness and death, it alone cannot achieve health equity. COVID-19 vaccines have demonstrably reduced hospitalizations, severe outcomes, and deaths, especially among vulnerable groups such as older adults and those with chronic diseases, thereby helping to prevent a syndemic situation [7].

However, the benefits of vaccination are not always equitably distributed, as social, economic, and geographic barriers can limit access for the most at-risk populations; therefore, integrating vaccination into routine healthcare and primary care services increases accessibility, particularly in resource-limited or remote settings. Primary care providers from each health region play a pivotal role in delivering vaccines, countering misinformation, and building trust within communities. This is largely due to their ability to coordinate with other social programs, such as the Family Grant Program (*Bolsa Família*) and the Street Clinic Program (*Consultório na Rua*), which help reach more people and ensure better access to health services.

Health regions are a key strategy for advancing healthcare, promoting justice, equity, and comprehensive care. These regions are defined not only by material and economic resources but are fundamentally shaped by shared values and guiding principles. To fully realize the potential of health regions—and thereby reduce inequalities in COVID-19 mortality—it is essential to foster changes in institutional culture and ensure ongoing professional training anchored in the principles of the SUS and primary healthcare.

This study advances knowledge by demonstrating COVID-19 mortality patterns across health regions. However, future studies could enhance understanding by stratifying data by age group, vaccine type, and comorbidity status, and by conducting interrupted time series analyses. Such approaches would provide a more precise assessment of vaccination effects and pandemic containment policies across São Paulo’s diverse regional contexts.

## 5. Conclusions

This study showed that COVID-19 mortality in the state of São Paulo is shaped by a multifaceted interplay of structural determinants, including the burden of chronic non-communicable diseases and the demographic composition of the population. The use of GAMLSS modeling enabled a nuanced analysis of these relationships, revealing that higher mortality from chronic diseases and lower proportions of adults were significantly associated with increased COVID-19 deaths. Moreover, regional disparities identified in the mortality trends across DRS highlight the role of territorial inequalities in shaping the pandemic’s impact.

Given the evidence presented, we recommend that future pandemic response efforts in Brazil and similar contexts adopt an integrated, territorially sensitive approach. This should combine disease surveillance, chronic disease management, equitable vaccine distribution, and social protection measures. Strengthening the regional capacity of the SUS remains essential for ensuring that no population is left behind in health emergencies.

## Figures and Tables

**Table 1:** Study variables categorized by dimension and data source.

Dimension	Variables	Date	Data Source
COVID-19	COVID-19 mortality rate per 100,000 inhabitants	2020–2021	SEADE
Health indicators	Applied doses (number of COVID-19 vaccines applied in the Population including unique, 1st and 2nd doses)	2021	DATASUS
	Deaths from non-communicable diseases (NCDs)	2020–2021	DATASUS
Social Indicators	General population	2020–2021	IBGE
	Population by age group	2020–2021	SEADE
	Urban population	2020–2021	IBGE
	Gini index	2020–2021	DATASUS

**Table 2:** Descriptive analysis of dependent and independent variables (February 2020–December 2021).

	Min	Median	Average	Max
Mortality rate per 100,000 inhabitants	0	7.00	15.10	290.60
Deaths from chronic non-communicable diseases (% of total population)	0	311.00	306.47	996.00
Percentage of population between 0 and 14 years old (% of total population)	7.40	19.84	21.31	30.49
Percentage of population aged 15–60 (% of total population)	57.09	65.38	68.91	80.43
Percentage of population aged 61 or older (% of total population)	9.30	16.22	16.42	31.47
Percentage of urban population (% of total population)	24.91	93.54	89.33	100
Gini index (range 0–1)	0.005	0.473	0.4586	0.6858
COVID-19 Vaccination	0.10	3747.00	18,023.61	4,314,565.00

**Table 3:** Final model adjusted to identify factors associated with mortality from COVID-19 in the state of São Paulo.

Μ	Estimate	Std. Error	t Value	Pr (>|t|) *	Relative Increase (%)
(Intercept)	3.128	0.14455	21.64	**<0.0001**	-
V2	0.08273	0.03094	2.674	**<0.0001**	8.62
V4	−0.19341	0.08952	−2.161	**0.0309**	−17.59
V5	−0.05353	0.06969	−0.768	0.4426	-
V9	0.01876	0.05132	0.365	0.7148	-
V10	−0.06153	0.0336	−1.831	0.0674	-
V11	0.13559	0.14247	0.952	0.3414	-
cod_drs_2	0.41721	0.24499	1.703	0.0889	-
cod_drs_3	0.04929	0.17025	0.29	0.7722	-
cod_drs_5	0.29798	0.19087	1.561	0.1188	-
cod_drs_6	0.34907	0.17454	2	**0.0457**	41.77
cod_drs_7	0.51329	0.1794	2.861	**0.0043**	67.08
cod_drs_8	0.12737	0.20243	0.629	0.5293	-
cod_drs_9	−0.36026	0.26183	−1.376	0.1691	-
cod_drs_10	0.01763	0.16375	0.108	0.9143	-
cod_drs_11	0.04877	0.19551	0.249	0.8030	-
cod_drs_12	0.15125	0.16585	0.912	0.3620	-
cod_drs_13	−0.13574	0.23555	−0.576	0.5645	-
cod_drs_14	0.27366	0.21084	1.298	0.1946	-
cod_drs_15	0.42503	0.19895	2.136	**0.0329**	52.96
cod_drs_16	0.36509	0.23625	1.545	0.1226	-
cod_drs_17	−0.08403	0.18163	−0.463	0.6437	-

* *p*-value (bolded to indicate significance, <0.05). Note: V2 = Deaths from non-transmissible chronic diseases; V4 = Percentage of population aged 15–60 years; V5 = Percentage of population aged 61+ years; V9 = Percentage of urban population; V10 = Gini index (World Bank calculation estimate divided by 100); V11 = COVID-19 vaccination (calculation of total applied doses of unique first and second vaccine doses); cod_drs_2—DRS II Araçatuba; cod_drs_3—DRS III Araraquara; cod_drs_5—DRS V Bauru, cod_drs_6—DRS VI Campinas; cod_drs_7—DRS VII Campinas; cod_drs_8—DRS VIII Franca; cod_drs_9—DRS IX Marília; cod_drs_10—DRS X Piracicaba; cod_drs_11—DRS XI Presidente Prudente; cod_drs_12—DRS XII Registro; cod_drs_13—DRS XIII Ribeirão Preto; cod_drs_14—DRS XIV São João da Boa Vista; cod_drs_15—DRS XV São José do Rio Preto; cod_drs_16—DRS XVI Sorocaba; cod_drs_17—DRS XVII Taubaté.

## Data Availability

This study utilized publicly available data from SEADE [9] (COVID-19 data), IBGE [7] (census data), and DATASUS [10] (vaccination data) to support the analysis.

## Abbreviations

The following abbreviations are used in this manuscript:

IBGE	Brazilian Institute of Geography and Statistics
GAMLSS	Generalized Additive Model for Location, Scale, and Shape
DRS	*Departamentos Regionais de Saúde*
NCDs	Non-communicable diseases
SEADE	State System Foundation for Statistical Data Analysis
SUS	*Sistema Único de Saúde*
DATASUS	Department of Information and Health Informatics of SUS
GAIC	Generalized Akaike Information Criterion
AIC	Akaike Information Criterion
